# 

**Published:** 1889-03

**Authors:** 


### BOOKS RECEIVED.

Essentials of Surgery. By Edward Martin, A.M., M.D.

Essentials of Anatomy. By Charles B. Nancrede, M.D.

Essentials of Medical Chemistry. By Lawrence Wolff, M.D.

Essentials of Obstetrics. By William Easterly Ashton, M.D.

The Waters of Plombiènes, Vosges. By Dr. Battentuit.

Clinical Lectures on Albuminuria. By Thomas Grainger Stewart, M.D., Edinburg.

Clinical Atlas of Venereal and Skin Disease. Parts III. and IV. By Robert W. Taylor, M.D.

The Vest-Pocket Anatomist. By C. Henri Leonard, M.D.

Transactions of the Association of American Physicians, Vol. III.

The Pathology and Treatment of Displacements of the Uterus. By Dr. B. S. Schultze.

The Annual Report of the Supervising Surgeon General of the Marine Hospital Service, 1888.

Guy's Hospital Reports, Vol. XLV.

Hand-Book of Materia Medica, Pharmacy and Therapeutics. By Cuthbert Bowen, M.D., B. A.

Wood's Medical and Surgical Monographs. Vol. I., Nos. 1 and 2.

Exploration of the Chest in Health and Disease. By Stephen Smith Burt, M.D.

International Pocket Medical Formulary.

Volume LVIII.] MARCH, 1889. [Number 3.

### The CHICAGO MEDICAL JOURNAL AND EXAMINER

EDITOR:
S. J. JONES, M.D., LL.D.

COLLABORATORS:

CHICAGO:

Professor E. ANDREWS, M.D., LL.D.
Professor J. BARTLETT, M.D.
Professor W. T. BELFIELD, M.D.
Professor F. BILLINGS, M.D.
Professor W. H. BYFORD, A.M., M.D.
Professor L. CURTIS, A.M., M.D.
Professor N. S. DAVIS, Jr., A.M., M.D.
Professor O. C. DE WOLF, A.M., M.D.
Professor C. FENGER, M.D.
Professor J. N. HYDE, A.M., M.D.
Professor E. F. INGALS, A.M., M.D.
Professor F. S. JOHNSON, A.M., M.D.
Professor H. A. JOHNSON, M.D., LL.D.
Professor C. W. PURDY, M.D.
Professor C. G. SMITH, A.M., M.D.
Professor E. WING, A.M., M.D.
Professor E. C. DUDLEY, A.B., M.D.

MILWAUKEE, WIS.:

Professor N. SENN, M.D., Ph.D.

WASHINGTON, D. C.:
S. O. RICHEY, M. D.

UNITED STATES ARMY:
Surgeon D. L. HUNTINGTON, M.D.

UNITED STATES NAVY:
Surgeon-General J. M. BROWNE, M.D. Medical-Director A. L. GIHON, A.M., M.D.

MARINE HOSPITAL SERVICE:
Surgeon S. T. ARMSTRONG, M.D., Ph.D.

